# Ultracentrifugation versus kit exosome isolation: nanoLC–MS and other tools reveal similar performance biomarkers, but also contaminations

**DOI:** 10.4155/fsoa-2018-0088

**Published:** 2018-11-09

**Authors:** Frøydis Sved Skottvoll, Henriette Engen Berg, Kamilla Bjørseth, Kaja Lund, Norbert Roos, Sara Bekhradnia, Bernd Thiede, Cecilie Sandberg, Einar Osland Vik-Mo, Hanne Roberg-Larsen, Bo Nyström, Elsa Lundanes, Steven Ray Wilson

**Affiliations:** 1 Department of Chemistry, University of Oslo, Post Box 1033, Blindern, NO-0315 Oslo, Norway; 2 Department of Microbiology, Unit Cell Signaling, Oslo University Hospital, Gaustadalleen 34, NO-0372 Oslo, Norway; 3 Department of Biosciences, University of Oslo, Post Box 1066, Blindern, NO-0316 Oslo, Norway; 4 Institute for Surgical Research & Department of Neurosurgery, Vilhelm Magnus Laboratory of Neurosurgical Research, Oslo University Hospital, 4950 Nydalen, NO-0424 Oslo, Norway; 5 Institute of Clinical Medicine, Faculty of Medicine, University of Oslo, Post Box 1171, Blindern, 0318 Oslo, Norway

**Keywords:** bioanalysis, exosomes, isolation

## Abstract

**Aim:**

For isolation of exosomes, differential ultracentrifugation and an isolation kit from a major vendor were compared.

**Materials & methods:**

‘Case study’ exosomes isolated from patient-derived cells from glioblastoma multiforme and a breast cancer cell line were analyzed.

**Results:**

Transmission electron microscopy, dynamic light scattering, western blotting, and so forth, revealed comparable performance. Potential protein biomarkers for both diseases were also identified in the isolates using nanoLC–MS. Western blotting and nanoLC–MS also revealed negative exosome markers regarding both isolation approaches.

**Conclusion:**

The two isolation methods had an overall similar performance, but we hesitate to use the term ‘exosome isolation’ as impurities may be present with both isolation methods. NanoLC–MS can detect disease biomarkers in exosomes and is useful for critical assessment of exosome enrichment procedures.

## Lay abstract

Exosomes are small vesicles that are released from biological cells. Exosomes are viewed as being tools for intracellular communication, and there is evidence that cancer cells can release exosomes to, for example, enable metastasis. Exosomes may be very useful for diagnostics, as the detection and characterization of exosomes may allow disease detection at early stages. However, exosomes must be isolated from blood or other biomaterials prior to analysis. This can be a challenging task, and we have here taken a critical look at two familiar isolation techniques, using samples from breast cancer cell lines and brain cancer cells (glioblastoma). We find that the two approaches have comparable performance, and contain protein biomarkers of their cells of origin. However, the isolated exosomes can contain contaminations, which may cloud the analysis in clinical settings. Hence, there is room for new approaches for the isolation of exosomes.

## Graphical abstract

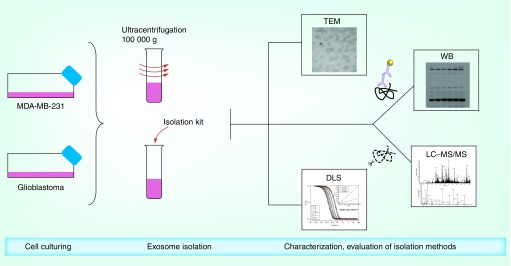

Exosomes are extracellular vesicles (EVs) that are secreted from cells to the extracellular environment as a part of the endocytic pathway [1]. The exosomes are formed by invagination of an endosome membrane to create intraluminal vesicles inside the endosome, in other words, multivesicular bodies, and are secreted when the endosomes fuse with the plasma membrane [2]. Exosomes commonly contain proteins originating from the cellular cytosol and the plasma membrane, nucleic acids (e.g. DNA, mRNA, miRNA and noncoding RNA), lipids and metabolites [1,3–8], and are believed to take part in, for example, cell–cell communication, transfer of proteins/nucleic acids, coagulation and antigen presentation [6,9].

Cancer cells have been found to release more exosomes than stromal cells, [10,11] and exosomes are associated with metastasis and tumor progression [7,12,13]. Hence, cancer exosomes may be a source of biomarkers for diagnosing cancers such as breast cancer (BC) and glioblastoma multiforme (GBM) when, for example, isolated from body fluids. BC is the predominant type of female cancer [14], with recurrent metastatic disease being responsible for the majority of BC-caused deaths [15]. GBM is the most frequent and malignant form of brain cancer [16–18]. The diagnosis of both BC and GBM rely on highly invasive patient tissue biopsies at relatively late stages [16,19,20]. Thus, a noninvasive disease monitoring is desirable for both BC and GBM, and can be achieved by measuring biomarkers in accessible body fluids, such as blood (liquid biopsy), for early diagnosis and prognosis assessment [16,21–23]. Hence, the isolation of exosomes for cancer biomarker discovery has emerged as an alternative to invasive methodologies [23–31].

Isolation of exosomes is predominantly performed from body fluids (e.g., blood, urine and saliva), or cell culture media by centrifugation-based methods, for example, sucrose density-gradient centrifugation or ultracentrifugation (UC) [32,33]. However, common drawbacks of using UC-based exosome isolation methods are the large amounts of starting material needed, low yield and poor reproducibility [34,35]. Moreover, there is a great need for exosome isolation protocols tailored toward smaller starting volumes for, for example, miniaturized cell culture models like organoids and ‘organ-on-a-chip’ [36,37]. Other exosome isolation protocols and principles have been developed to overcome the drawbacks of UC-based methods. Among these, filtration, immunoaffinity capturing, size exclusion chromatography, flow field-flow fractionation and also acoustic trapping have been attempted [8,34,38–44]. In addition, different commercial exosome isolation kits are available (e.g., ExoQuick™ from Systems Biosciences, CA, USA, and Total Exosome Isolation™ from Thermo Fisher Scientific, MA, USA), commonly known to precipitate exosomes with water-excluding polymers such as polyethylene glycol [45]. These isolation kits enable simple isolation of exosomes from small starting volumes from a wide range of matrices. However, these kits are arguably less studied and understood compared with UC approaches, which are arguably sources of skepticism.

A previous study has compared commercial isolation kits and UC, with emphasis on exosomal RNA [46], while we here place emphasis on untargeted proteomics using nano-liquid chromatography coupled with mass spectrometry (nanoLC–MS) for our samples. The methods were additionally evaluated using the following characterization techniques: western blotting (WB), transmission electron microscopy (TEM), dynamic light scattering (DLS) and quantitative total protein analysis using UV–vis spectrophotometry. ‘Case study’ exosomes were isolated from cell culture media from free-floating, patient-derived primary cell cultures from GBM biopsies (T1018) and a serum cultivated, adherently growing BC cell line (MDA-MB-231).

## Materials & methods

### MDA MB-231 cell culturing

The BC cell line was purchased from American Type Culture Collection (ATCC, Sesto San Giovanni, Milan, Italy), and is derived from a triple-negative human metastatic breast carcinoma. The cells were maintained in Rosewell Park Memorial Institute 1640 growth medium depleted of phenol red (Sigma–Aldrich, MO, USA) supplemented with 10% exosome-depleted fetal bovine serum (FBS, centrifugation-based exosome depletion, Systems Biosciences) and 1% penicillin/streptomycin (Sigma–Aldrich). The cells were incubated in a humidifying atmosphere at 5% CO_2_ and at 37°C. Prior to exosome isolation, 1–2.3 million cells (in T75-T175 culturing flasks) were incubated for 6–7 days (always using a passage lower than 12). The incubated cell culture medium was centrifuged at 906 g (30 min at 23°C; Supplementary Methods, section S-1).

### Glioblastoma cell culturing

The GBM cells (T1018) were derived from biopsies from a primary GBM tumor, obtained after informed consent through a biobank approved by the Regional Ethical Authorities operated at Oslo University Hospital (2016/1791). The cells were maintained in Dulbecco's modified eagle medium with nutrient mixture F-12 (DMEM/F12, Gibco, Thermo Fisher Scientific), supplemented with HEPES buffer (10 mM) and penicillin/streptomycin (100 U/ml) from Lonza (Basel, Switzerland), B27 without vitamin A (1/50) from Thermo Fisher Scientific, epidermal growth factor (20 ng/ml) and basic fibroblast growth factor (10 ng/ml) from R&D Systems (MN, USA) and heparin (2.5 μg/ml) obtained from LEO Pharma AS (Ballerup, Denmark). Under these culturing conditions, cells express stem cell markers *in vitro*, differentiate upon removal of growth factors and give rise to diffusely infiltrative tumors upon xenografting [47]. The cells were incubated in a humidifying atmosphere at 5% CO_2_ and 37°C in T25 flasks (Thermo Fisher Scientific). Prior to exosome isolation, the incubated cell culture medium was centrifuged twice at 453 g and 1811 g for 5 min each (Supplementary Methods, section S-1).

### Exosome isolation by ultracentrifugation

For the BC and GBM cells, 9–12 and 60 ml cell culture media were used for centrifugation. Cell culture media were first centrifuged at 1811 g (5 min at 4°C). The supernatants were then centrifuged at 20,000 g (20 min at 4°C) with an Allegra 25R centrifuge (with TA-14-50 rotor) from Beckman Coulter (CA, USA), and the supernatants were transferred to polycarbonate UC tubes (Beckman Coulter) and diluted with phosphate-buffered saline (PBS; ∼60 ml in each). The tubes were centrifuged twice at 100,000 g (90 min at 4°C) with an L-80 ultracentrifuge (45 Ti rotor) from Beckman Coulter. The supernatants were removed (leaving suspension 1 cm above the pellets), and the pellets were suspended with PBS between the centrifugations. Upon centrifugation, the supernatants were discarded, and the exosome pellets (UC isolates) were suspended in either PBS (3 ml for DLS- and 50–100 μl for TEM analysis) or the preferred lysis buffer.

### Exosome isolation by isolation kit

The isolation of exosomes with the kit was performed with the Total Exosome Isolation Reagent (from cell culture media) from Thermo Fisher Scientific (catalog number 4478359). The isolation was performed according to the protocol of the supplier. Starting volumes ranged from 0.5 to 9 ml cell culture medium for the BC cells, and 5 to 6 ml for the GBM cells. The samples were centrifuged with the Allegra 25R centrifuge, and the exosome pellets (kit isolates) were suspended as with UC.

### Protein extraction

Cell and exosome protein extracts were made by lysis with RIPA- or Nonidet™ P40 (NP40) buffer (both from Thermo Fisher Scientific) containing protease inhibitors (Protease Inhibitor Cocktail Tablets, Roche, Basel, Switzerland) and phosphatase inhibitors (PhosStop Tablets, Sigma–Aldrich; Supplementary Methods, section S-2). The protein amount was measured using Pierce™ BCA protein Assay Kit (Thermo Fisher Scientific), by measuring the absorbance at 562 nm (Supplementary Methods, section S-3).

### Western blotting

For information about WB antibodies, procedures and equipment, see Supplementary Methods, section S-4.

### Transmission electron microscopy

Samples were visualized with a JEM-1400Plus transmission electron microscope from JEOL (Tokyo, Japan) and images were recorded at 80 kV (Supplementary Methods, section S-5).

### Dynamic light scattering

The DLS experiments were conducted with the aid of an ALV/CGS-8F multidetector version compact goniometer system, with eight fiber-optical detection units, from ALV-GmbH, Langen, Germany. See Supplementary Methods, section S-6 for more details. See also the Supplementary DLS Data file.

### NanoLC–MS analysis

NanoLC–MS was performed using Q-Exactive mass spectrometers (Thermo Fisher Scientific) coupled with nano-liquid chromatography. Samples were prepared by in-solution and in-gel protease digestion. Database searches were performed using both SEQUEST and MASCOT algorithms. Peptides were identified with high peptide confidence filter (>99% confidence) with a false discovery rate threshold of ≤0.01. One signature peptide was selected as a requirement for protein identifications during a database search. See Supplementary Methods, section S7–S9 for additional information related to nanoLC–MS analysis.

## Results & discussion

### TEM & DLS detected vesicles in the expected size range for exosomes

**Figure 1.**
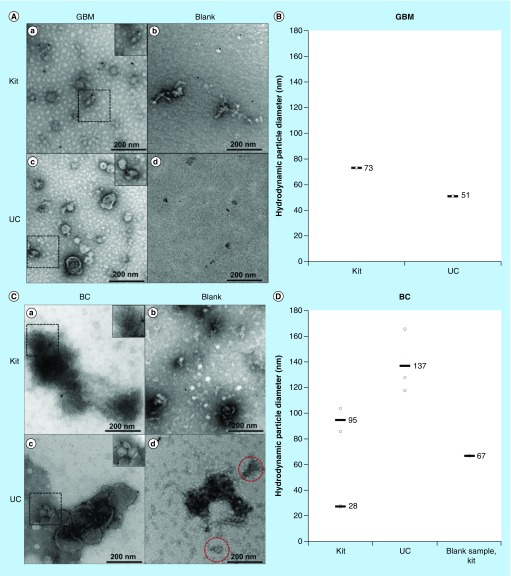
**Transmission electron micrographs and hydrodynamic particle size (nm) distribution by dynamic light scattering analysis of exosomes isolated by kit and ultracentrifugation from glioblastoma multiforme and  breast cancer cells.** Images were taken with a magnification of 400,000 and the dashed areas were additionally zoomed. **(A)** Micrographs of GBM exosome isolates (not CD9-labeled). **(a)** depicts the micrograph from a kit isolate; **(b)** the kit blank; **(c)** a UC isolate; and **(d)** the UC blank. **(B)** DLS analysis of GBM exosomes isolated by kit and UC. No particles were detected in the UC blank. The DLS analysis of the kit blank was not performed. **C**) Micrographs of BC exosome isolates (successfully CD9-labeled). **(a)** depicts the micrograph from a kit isolate; **(b)** the kit blank; **(c)** a UC isolate; and **(d)** the UC blank. **(D)** DLS analysis of BC exosomes isolated by kit and UC, including the kit blank. No particles were detected in the UC blank. BC: Breast cancer; DLS: Dynamic light scattering; GBM: Glioblastoma multiforme; UC: Ultracentrifugation.

Morphological analysis of the exosome samples was performed using TEM. In addition, the hydrodynamic particle size distribution was measured using DLS. Clusters of vesicles were observed in the micrographs of the samples isolated with both kit and UC (Figure 1AI & AIII). Vesicle structures similar to that described in literature were observed [6,48,49]. Regarding GBM exosomes: with TEM, the UC isolates presented somewhat more distinct double membranes compared with the kit isolates. The blank samples for both isolation methods did not display membrane structures (Figure 1AII & AIV). The DLS-analysis of the GBM isolates exhibited particles of similar sizes of 51 and 73 nm (mean) with both isolation methods (Figure 1B). Thus, both isolation methods gave rise to comparable exosome populations. Regarding BC exosomes: clusters of vesicles were also in here observed in the micrographs of the samples isolated with both kit and UC (Figure 1CI & CIII). Blank isolates displayed contaminations (Figure 1CII & CIV), for example, exosome-resembling vesicles were found in the UC blank using TEM (red dashed circles), and the kit blank displayed 67 nm (mean) contaminations when using DLS (Figure 1D). Somewhat surprisingly, the DLS analysis also presented two distinct particle diameter means in kit isolates (28 and 95 nm, mean values), while only one particle diameter was present in UC isolates (137 nm, mean value), indicating differences in the mean particle sizes isolated with the two isolation methods. However, the sizes observed with DLS correlate with that found in other studies (30–250 nm) [13,50–54]. Overall, the isolates showed structures resembling those of EVs, but some blanks were not entirely devoid of vesicles or particles.

### WB analyses indicated the presence of exosomes for all samples

**Figure 2.**
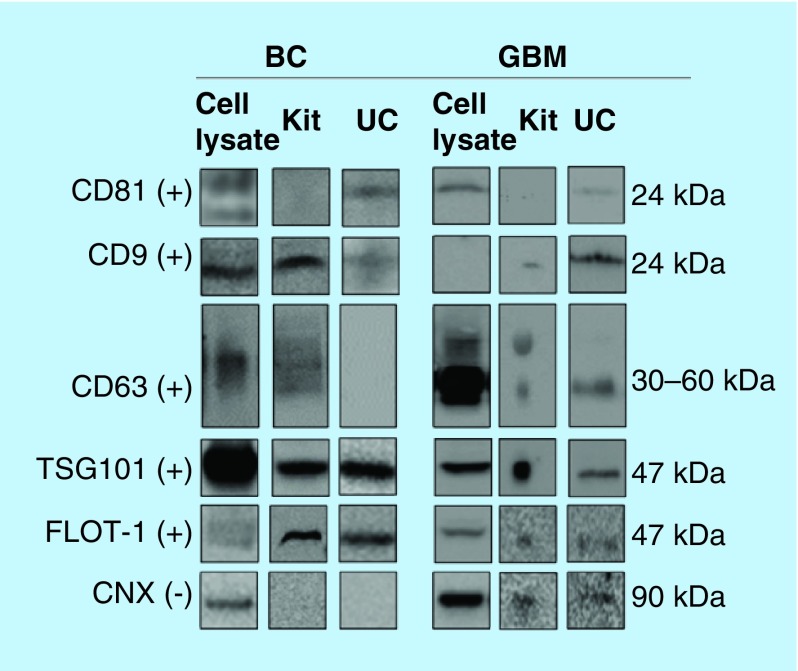
**Western blotting of common protein exosome markers.** The protein markers CD81, CD9, CD63, TSG101, FLOT1 (positive markers, +) and CNX (negative marker, -) were targeted in cell lysates, and exosomes isolated by kit and UC (n ≥ 2). Monoclonal mouse antibodies were used for CD81, CD9, CD63, FLOT1 and CNX, while a polyclonal rabbit antibody was used for TSG101. For the BC exosomes, 15 μg protein was loaded for kit isolates and 3 μg for UC isolates. For the GBM exosomes, approximately 14 μg was loaded for kit isolates and approximately 8 μg for UC isolates. Uncropped western blots are presented in Supplementary western blots. BC: Breast cancer; CNX: Calnexin; FLOT1: Flotillin-1; GBM: Glioblastoma multiforme; UC: Ultracentrifugation.

The WB was performed using antibodies for a selection of positive exosome markers, namely the tetraspanins CD81, CD9 and CD63, TSG101 and FLOT1. CNX was selected as a negative marker for purity evaluation as recommended by the International Society of Extracellular vesicles [55]. This protein is located at the endoplasmic reticulum, and is assumed to signalize ER-contamination. For the GBM cells and exosomes, positive markers CD63, CD9, TSG101 and FLOT1 were detected in isolates from both the kit and UC (Figure 2). The positive marker CD81 was not detected in isolates from kit. However, the negative exosome marker CNX was detected in both GBM cells and exosomes using both isolation methods. For the BC cells and exosomes, positive markers TSG101, FLOT1 and CD9 were detected using both isolation methods, and CNX was not detected. Some interesting differences were noticed, perhaps due to the isolation technique; for example, CD81 was not detected using kit isolation, which we could not explain. The positive markers overall demonstrate the presence of exosomes in the isolates obtained using both methods, but the GBM samples could contain impurities. However, higher purity of the samples could possibly be achieved by increasing the cycles of UC [56]. Regarding total protein amounts; when subtracting the measured in blank samples from the protein amount in exosome isolates, the measured protein content for exosomes isolated by the kit and UC was similar (measuring absorbance at 562 nm after BCA reaction).

### NanoLC–MS studies reveal impurities & protein biomarkers

**Figure 3.**
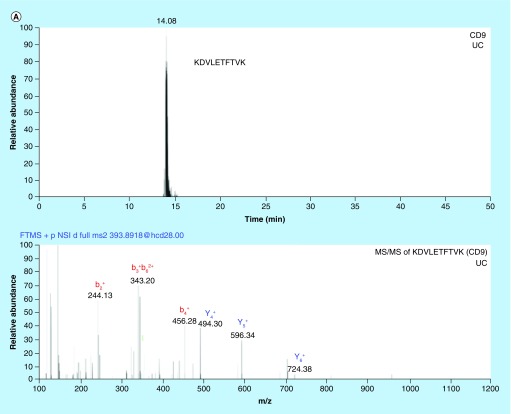

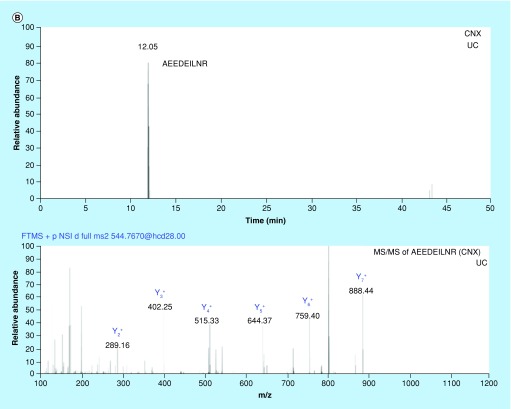
**Chromatograms and MS/MS spectrums from nanoLC–MS analysis of in-gel digested glioblastoma multiforme and breast cancer exosome peptides using data-dependent acquisition.** **(A)** Chromatogram with corresponding MS/MS spectrum for the CD9 signature peptide KDVLETFTVK (*m/z* = 393.89, z = 3) in BC exosomes isolated by UC. **(B)** Chromatogram with corresponding MS/MS spectrum for the CNX signature peptide AEEDEILNR (*m/z* = 544.77, z = 2) from GBM exosomes isolated by UC. An in-house packed 50 μm × 150 mm column with 80 Å Accucore particles with C_18_ stationary phase was used for separation. The elution was performed with a linear gradient of 3–15% MP B in 120 min. BC: Breast cancer; GBM: Glioblastoma multiforme; UC: Ultracentrifugation.

Untargeted nanoLC–MS was performed with samples prepared by in-solution and in-gel protease digestion. In-house prepared nanoLC columns packed with core shell particles enabled high-resolution separations (Figure 3, see reference [57] for packing procedure).

**Figure 4.**
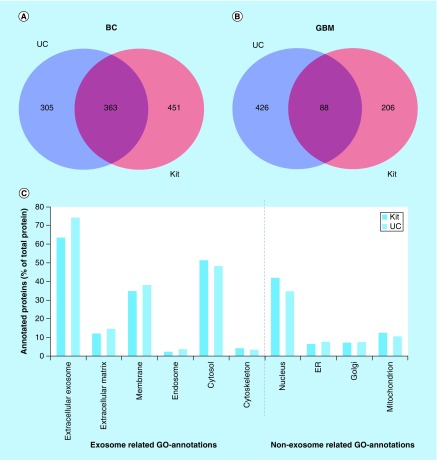
**Venn diagram comparing the number of proteins identified by nanoLC–MS/MS in glioblastoma multiforme and breast cancer exosome samples and gene ontology annotation of proteins in breast cancer exosomes.** **(A)** Venn diagram of proteins identified in BC exosome samples with UC (from 2697 identified peptides) and kit (from 3795 peptides identified) (n = 3). **(B)** Venn diagram of proteins identified in GBM exosome samples with UC (from 1840 identified peptides) and kit (from 1035 identified peptides; n = 6). **(C)** GO annotation of proteins in BC exosomes. The identified proteins were classified by their cellular location (GO annotations) and then grouped based on their positive/negative relevance toward exosomes. The annotated proteins (% of total proteins) and their cellular location from kit isolates (red, from 749 DAVID ID's) and UC isolates (blue, from 615 DAVID IDs). BC: Breast cancer; GBM: Glioblastoma multiforme; GO: Gene ontology; UC: Ultracentrifugation.

When comparing cell sources (Figure 4A & B), the number of identified proteins was lower in GBM isolates than BC isolates, but the number of identified proteins for GBM isolates was comparable with another nanoLC–MS study on GBM exosomes [58]. See also Figure 4A and B (Venn diagram) illustrating the overlaps/differences in identified proteins. Even though the absence of CNX (detected with WB) in BC exosomes from both isolation methods indicated that the isolates were not contaminated with the ER, untargeted nanoLC–MS suggested the presence of impurities also in the BC samples; general proteins related to, for example, the nucleus, Golgi apparatus, mitochondrion and ER were identified in the BC exosomes using gene ontology (GO) annotations (Figure 4C). Proteins related to the nucleosome, Golgi apparatus, mitochondrion, and ER were also identified by GO-annotation in the GBM isolates (results not shown).

NanoLC–MS could also identify a number of positive markers for exosomes. However, there was expectedly not a complete overlap with those observed with WB, as, for example, sensitivity can vary between WB and untargeted LC–MS, depending on the analyte, ion suppression, and so forth. Examples of potential biomarkers for GBM disease, for example, HSP70 and HSP90 [59–61], CSPG4 [59,62], CD44 [59,62,63] and CD276 [64] were identified in UC isolates but not in kit isolates using untargeted nanoLC–MS. Examples of LC–MS detected biomarkers related to triple negative BC were, for example, HSP90A and HSP90B [65], CALM1 and EGFR [66] (detected with both methods, see Supplementary Proteins).

## Conclusion

Testing with a limited number of glioblastoma/BC ‘case study’ samples, the UC/kit isolation methods overall were arguably equal in quality. Kit isolation, however, has an advantage of requiring less starting material compared with conventional UC equipment. Untargeted nanoLC–MS revealed a number of biomarkers related to the diseases, supporting the concept of exosomes being an interesting matrix toward diagnostics. In addition to exosomes, our analyses suggest the presence of cellular contaminations and other vesicles. Hence, the ‘isolations’ should perhaps be considered ‘enrichments’.

## Future perspective

Considering that the methods do not fully provide isolations, we believe that alternative approaches to preparing and analyzing these important EVs will emerge as standards. Moreover, the methods investigated cannot distinguish between exosomes (healthy vs cancer); to exploit the possibilities of nanoLC–MS for unraveling cancer biomarkers in exosomes, additional steps must be taken, for example, sorting exosomes according to surface markers associated with various cancers. Such approaches are being investigated [67], and combining exosome sorting with nanoLC-MS can be a powerful tool in the future.

**Summary points**

- Using ‘case study’ exosomes from brain cancer cells/breast cancer cell line, ultracentrifugation and a commercial kit provide comparable performance for isolating exosomes.
- Biomarkers readily identified in exosomes using nanoLC–MS.
- Contaminations detected in exosome samples, encouraging additional methods for isolations to be developed and standardized.
