# Supplementary material for: Ultracentrifugation versus kit exosome isolation: nanoLC–MS and other tools reveal similar performance biomarkers, but also contaminations
Source: Future Sci OA. 2018 Nov 9;5(1):FSO359. doi: 10.4155/fsoa-2018-0088 (PMC6331754; doi:10.4155/fsoa-2018-0088)
Supplement: Supplementary file 1 [file fsoa-05-359-s1.docx]

# Supplemental DLS data

**(AUTHOR INFORMATION TO BE PLACED HERE AFTER REVIEW)**

All figures are correlation functions from DLS analyses of isolated exosome samples. Table 1 shows an overview of the figure number related to the sample code of interest, together with the measured hydrodynamic radius in the samples.

Table 1. Figure number of the related correlation function to the different sample codes, together with the measured hydrodynamic radius (nm).

| **Figure #** | **Sample code** | **Hydrodynamic radius (fast) in nm.** | **Hydrodynamic radius (slow) in nm.** |
| --- | --- | --- | --- |
| **Figure 1** | **BC UC 1** | **83.13** | **--** |
| **Figure 2** | **BC UC 2** | **64.1** | **--** |
| **Figure 3** | **BC UC 3** | **59.18** | **--** |
| **Figure 4** | **BC kit 1** | **13.5** | **52.1** |
| **Figure 5** | **BC kit 2** | **13.9** | **43.1** |
| **Figure 6** | **GBM UC 1** | **25.6** |  |
| **Figure 7** | **GBM kit 1** | **36.7** |  |

Figure 1. Correlation function of BC UC replicate 1.

Figure 2. Correlation function of BC UC replicate 2.

Figure 3. Correlation function of BC UC replicate 3.

|   Figure 4. Correlation function of BC kit replicate 1. |
| --- |
|   Figure 5. Correlation function of BC kit replicate 2. |

Figure 6. Correlation function of GBM UC replicate 1.

Figure 7. Correlation function of GBM kit replicate 1.
