# Supplementary material for: Ultracentrifugation versus kit exosome isolation: nanoLC–MS and other tools reveal similar performance biomarkers, but also contaminations
Source: Future Sci OA. 2018 Nov 9;5(1):FSO359. doi: 10.4155/fsoa-2018-0088 (PMC6331754; doi:10.4155/fsoa-2018-0088)
Supplement: Supplementary file 2 [file fsoa-05-359-s2.docx]

Supplemental Methods

**(AUTHOR INFORMATION TO BE PLACED HERE AFTER REVIEW)**

Unless otherwise stated, water (commonly type 1 water purified by a Direct-Q® water purification system from Millipore (Billerica, MA, USA)) was used as solvent.

S-1 Cell culturing and exosome isolation preparation

All cell culture handling was performed sterile and liquids were pre-heated to ~37 ^o^C. A Forma^TM^ Steri-cycle^TM^ from Thermo Fisher Scientific, Waltham, MA, USA) was used for incubation of the BC cells. More information about the two cell lines applied in this study are shown in **Table S.1**.

Table S.1: Information about the two cancer cell lines applied in this study with their abbreviation, catalog number, type of organism and organ (disease).

| Cell line | Catalog number | Organism | Organ (disease) |
| --- | --- | --- | --- |
| MDA-MB-231 | HTB-26^TM^ | *Homo sapiens* | Mammary gland; breast (adenocarcinoma). Estrogen receptor negative. |
| T1018 |  | *Homo sapiens* | Brain. GBM. Primary. |

The amount of BC cells (cells/ mL cell culture media) was counted with a cell counter from Bio Rad (Hercules, CA, USA). 6 µL cell culture media with cells was used for counting. Prior to exosome isolation, the cell culture media was transferred to 15 mL tubes and spun down at 906 × *g* (30 minutes at 23 °C), and the supernatant was transferred to new 15 mL tubes and frozen at -20 ^o^C upon further use.

For the cell counting of GBM cells, Trypan Blue stain 0.4 % (Thermo Fisher Scientific) was used with a Countess Automated Cell Counter (Thermo Fisher Scientific). For exosome isolation, the remaining cell culture medium (after cell counting) was transferred to 50 mL tubes and centrifuged twice at 453 × *g* and 1811 × *g* for 5 min each. The supernatant was transferred to new 50 mL tubes before the second centrifugation.

An Eppendorf 5810/5810 R Centrifuge with an A-4-62 swing-bucket was used for the centrifugation for both cell lines.

S-2 Protein extraction

*S-2.1 Solutions*

The RIPA buffer was prepared by adding one tablet of both phosphatase inhibitor tablets and EDTA-free protease inhibitor tablets (both from Roche, Basel, Switzerland) to 10 mL of radioimmunoprecipitation assay (RIPA) lysis and extraction buffer (Thermo Fisher Scientific), vortexing until fully dissolved and kept on ice until use or stored in 1 mL aliquots (1.5 mL tubes from VWR) at -20 °C. RIPA was reusable when kept on ice and refrozen after use. For the in-house prepared NP40 buffer, 2.5 mL 1M 2-amino-2-(hydroxymethyl) propane-1,3-diol (tris) (pH 8, from MIK), 1.5 mL 5 M NaCl (99.995 % trace metals basis, Sigma), 1.5 mL NP40 detergent solution (Thermo Fisher Scientific, catalog number: 85124) and 200 µL EDTA (Sigma) were mixed in a 50 mL tube and diluted with 43.4 mL water. The solution was frozen at -20 °C in 10 mL aliquots. When needed, one 10 mL aliquot was thawed; one protease inhibitor tablet and phosphatase inhibitor tablet (both from Sigma) were added and vortexed until dissolved. The solution was further aliquoted into 1 mL tubes, which were stored at – 20 ℃ (stable for a few months). The NP40 buffer was reusable when kept on ice and refrozen after use.

*S-2.2 Lysis*

The BC exosome pellets from ultracentrifugation and isolation kit were dissolved in 50 µL PBS and 50 µL of the prepared NP40 buffer for lysis prior to LC-MS/MS analysis and the corresponding bicinchoninic acid (BCA) analysis. For WB and the corresponding BCA analysis (results not included), the BC exosomes were lysed in 50 µL RIPA buffer and the BC cells were lysed in 100 µL RIPA buffer. For the GBM exosome pellets, 300 µL RIPA buffer was added for WB and corresponding BCA analysis (results not included), while 1 mL RIPA buffer was added to the GBM cells. For LC-MS/MS and the corresponding BCA analysis, the remaining volume after TEM was dried using a SpeedVac C110 concentrator from Savant (now Thermo Fisher Scientific) at room temperature (RT) and lysed in 50 µL NP40 buffer. All suspensions were transferred to 1.5 mL tubes and rotated on a SB3 rotator from Stuart Equipment (Stone, Staffordshire, UK) or Invitrogen Hulamixer (Thermo Fisher Scientific) at 8 RPM (30 minutes, 4 ℃) and frozen (-80 ^o^C for the GBM samples, snap frozen using liquid nitrogen and stored in -20 ^o^C for the BC samples) until further use.

S-3 Determination of protein concentrations by UV-Vis spectrophotometry

Lysed samples were thawed and centrifuged at 20 570 × *g* (30 minutes at 2$℃$) using a Heraeus Fresco 21 centrifuge (Thermo Fisher Scientific) or an Eppendorf 5424R Centrifuge. The supernatant was transferred to new Eppendorf tubes and the pellet was disposed. Reagent A and B were mixed at a ratio of 50:1. For BCA-analysis of the BC-samples, 100 $\mu L$ of the mixture was distributed in a 96-microwell plate (VWR (BC) or Nunclon MicroWell plates from Sigma Aldrich (GBM)). The bovine serum albumin (BSA) calibration-standard following the BCA assay kit was distributed to the wells (triplicates) and diluted with PBS to a total volume of 110$\mu L$, yielding in seven wells with increasing amounts of BSA (0-7 $\mu g$). Next, mixing was performed by pipetting up and down. 10 $\mu L$ of the isolates were distributed to the wells as a duplicate or triplicate depending on the amount of sample available. The micro well plates were placed in an incubator holding 37.5 $℃$ for 30 minutes prior to the protein measurements. Measurements were performed at 562 nm on FLUOstar Omega microplate using a Wallac Victor2 1420 multilabel counter and Wallac 1420 Workstation software (version 3.00), both from Perkin Elmer (Waltham, MA, USA).

For the BCA analysis of GBM exosomes prior to LC-MS/MS analysis, only 10 µL of the BCA mixture was distributed and 1 µL of the isolates were added (11 µL in total) to two wells (duplicate). A Nanodrop 2000 Spectrophotometer from Thermo Fisher Scientific was used for the protein measurements.

S-4 Western Blot

*S-4.1 Solutions*

5x loading buffer was made by mixing 0.6 mL 1M Tris-HCl (pH 6.6), 1mL 1 % bromophenol blue (w/v), 2 mL 10 % sodium dodecyl sulphate (SDS), 0.5 mL 2-mercaptoetanol and 5 mL glycerol/water (50/50, v/v) (all from Sigma Aldrich) and 0.9 mL water. The mixture was vortexed and kept in 15 mL tubes at RT until use. Tris buffered saline (TBS) in polyoxyethylene (20) sorbitan monolaurate (Tween-20) was made by diluting 10 TBS-Tween-20 tablets from Medicago (Uppsala, Sweden) to 5 L with water (5 % TBS/Tween-20, hereafter called TBS-T). The buffer was stored at RT.

*S-4.2 Gel electrophoresis*

The protein extracts containing 3-15 µg protein were diluted with water giving equal protein concentrations before adding 5x loading buffer to yield 1x.

The samples were placed on a Grant QBT2 heating block (3 minutes at 92 $℃$) from Grant Instruments (Shepreth, Cambridgeshire, UK). 4-12 % 2-bis (2-hydroxyethyl) amino-2-(hydroxymethyl) propane-1,3-diol (Bis-Tris)) gels from Novex Thermo Fisher Scientific) were inserted to the midi or mini Cell electrophoresis chamber (both Thermo Fisher Scientific). The chamber was filled with the proper running buffer; 2-(N-morpholino) ethanesulfonic acid (MES) or 3-morpholinopropane-1-sulfonic acid (MOPS) SDS Running Buffer (1x).

The samples were distributed to the wells of the chamber (of volumes from 15-35$\mu L$) by using microcapillary tips pipettes (VWR). One well per gel was loaded with 5-10$\mu L$ PageRuler™ Prestained protein ladder (Thermo Fisher Scientific). A voltage of 70-75 V (150 mA) was applied to the chamber with the power supply (Bio-Rad, Hercules, CA, USA) for one hour, and increased to 110 V (150 mA) if the blue lane from bromophenol blue was horizontally distributed.

*S-4.3 Protein transfer*

The 10x transfer buffer was made by mixing 30.3 g Trizma® base (≥99 %) and 144.0 g glycine (≥99 %, HPLC) both from Sigma, diluted to 1000 mL with water. Prior to use, 100 mL of the 10x buffer were mixed with 200 mL technical methanol (MeOH, from VWR) and 700 mL water. Both buffers were stored at RT.

A 45 µm nitrocellulose membrane (Thermo Fisher Scientific) was cut to fit the size of the gel and both the membrane and the gel were separately incubated in the 1x transfer buffer for 15 minutes. Two Extra Thick Blot Filter Papers (Bio-Rad) were drenched with the 1x transfer buffer, and all four components were placed in the transfer chamber for semi-dry electroblotting (Bio-Rad) with the following layering order starting at the bottom; filter paper, membrane, gel, filter paper. At every layer, 1x transfer buffer was added to keep the components moisturized. In addition, the body of a 10 mL pipette was rolled over every layer to remove air bubbles between the layers and excess fluid outside the transfer sandwich was wiped away. 1 W was applied, and the transfer chamber was left over night at 4 ℃. The following day, the membrane was cut and the individual protein bands marked using a pencil.

*S-4.4 Immunolabelling and visualizing*

The transferred proteins were blocked in a solution of consisting of 5 % non-fat dry milk (PanReac AppliChem ITW reagents, Darmstadt, Germany) in 5 % TBS-T (milk/ 5 % TBS-T, 5/95, w/v) for 1 hour on a mixing plate. The mixture containing milk and TBS-T was discharged and the membranes were transferred to 50 mL tubes containing 5 mL of the corresponding primary antibody solutions with dilution factor (diluted with 5 % non-fat dry in TBS-T), listed in **Table S.2**. The tubes were kept on a roller from Stuart (Stone, Staffordshire, UK) over night at 4 ℃ before the membranes were removed and washed twice with 5 % non-fat dry milk in TBS-T for 10 minutes on a mixing plate. The membranes were transferred to new 50 mL tubes containing 5 mL of secondary antibody solutions reflecting the host animal of the primary antibody (e.g. anti-mouse, anti-rabbit, **Table S.2**), and the tubes were kept on a roller for 2 hours at RT. Both the tubes with the primary and the secondary antibody solution were cryopreserved and reused once.

**Table S.2: Overview of the primary and secondary antibodies applied in WB and TEM, with information about dilution factor (diluted with 5 % TBS-T**)**, clone if not polyclonal, host animal, catalog number and supplier.**

| Antibody | Dilution factor | Clone, if not polyclonal | Host animal | Catalog number | Supplier |
| --- | --- | --- | --- | --- | --- |
| CD9 | 1000 | TS9 and DRAP-27 | Mouse and rabbit | 10626D and PA5-11559 (TEM) | Thermo Fisher Scientific |
| CD63 | 500 (BC)/1000 (GBM) | TS63 | Mouse | 10628D | Thermo Fisher Scientific |
| CD81 | 500 | 1.3.3.22 and M38 | Mouse | MA5-13548 and 10630D | Thermo Fisher Scientific |
| TSG101 | 500 | - | Rabbit | T5701 | Sigma |
| flotillin-1 | 500 | 18/Flotillin-1 | Mouse | 610821 | BD Biosciences, San Jose, CA, USA |
| calnexin | 1000 | 37/Calnexin | Mouse | 610523 | BD Biosciences |
| actin | 1000 | - | Rabbit | A2066 | Sigma |
| Anti-mouse | 10 000 | - | Chicken | Sc-2954 | Santa Cruz |
| Anti-rabbit | 10 000 | - | Chicken | Sc-2955 | Santa Cruz |
| Anti-mouse |  | - | Rabbit | Z0259 | Dako, Glostrup, Denmark |

Next the membranes were further washed three times with TBS-T (30 minutes on a mixing plate). The fluid was replaced with equal amount of freshly mixed enhanced chemilumiscense prime (ECL-prime) (0.5 mL per membrane) from GE Healthcare (Buckinghamshire, UK) and pipetted from membrane to membrane for 3-5 minutes. Transparency films from Nobo (integrated part of ACCO Brands Corporation, Lake Zurich, IL, USA) were cut, and the membranes were put in between two sheets of the film with additional ECL-prime poured over the membranes. Bubbles in between the sheets were removed by “ironing” the film surface with thick filter paper. The bands were developed in a Chemidoc^TM^ touch imaging system from Bio-Rad. To visualize the bands, the proper size of the live view was chosen inside the developer, chemilumiscense was chosen as setting and the exposure time was set depending on the antibody. For the proteins actin, GAPDH, TSG-101 and flotillin-1, a short exposure time of 60 seconds was sufficient for visualization. Other proteins were exposed for 3000-5000 seconds. The raw files were processed with the Image Lab™ Software (version 6.0) from Bio-Rad.

S-5 Transmission electron microscopy

The copper grid (100 square mesh) from Electron Microscopy Sciences (Hatfield, PA, USA) was coated with formvar from Agar Scientific (Stansted, Essex, UK). The solutions of 4 % uranyl acetate (uranyl acetate/water, 4/96, w/v) and 1 % gelatin from cold water fish skin (FSG) (FSG/PBS, 1/99, v/v) were purchased from Sigma.

One drop of 5-50 $\mu L$ (depending on the amount of isolate available) of the lysed isolates was placed on clean Parafilm (VWR), and the formvar coated cobber grid was carefully placed to float on the drop with the coated side facing the suspension. The material was allowed to adsorb for 5-20 minutes, before rinsing on two large drops of PBS (approximately 1000$\mu L$) for 5 minutes followed by incubation on a drop (8$\mu L$) of the desired primary antibody diluted (anti-CD9, 1+9 with 1 % FSG) for 20 minutes.

Next the grids were washed on two large drops of PBS (approximately 1000$\mu L$) for 5 minutes. If bridging (i.e. secondary antibody) was necessary, the grid was incubated on a drop (5 $\mu L$) of rabbit anti-mouse antibody (diluted 1:200 with 1 % FSG) for 25 minutes, before repeating the washing on two drops of PBS (approximately 1000 $\mu L$) for 5 minutes. Bridging- and not bridging suspensions was both further incubated for 20 minutes on a drop of diluted protein A-gold-1 % FSG (1/50, v/v) (10 nm particles size), purchased from Cell Microscopy Core (CMC, University Medical Center Utrecht, Utrecht, The Netherlands). A final wash on 5 drops of PBS (2 minutes) followed by 5 drops of water (3 minutes) was performed before negative staining with 4 % uranyl acetate for 2 minutes. The excess fluid was blot by gently pushing the grid sideways on a filter Whatman^®^ qualitative filter paper (Sigma), and the grids were stored in a storage box at RT until use.

The grids were placed on a specimen quartet holder (EM-01070 SQH) and visualized with a JEM-1400Plus transmission electron microscope from JEOL (Peabody, MA, USA). Images were recorded at 80 kV and further image processing was done with Power Point software.

S-6 Dynamic light scattering

The beam from a Uniphase cylindrical 22 mW HeNe-laser, operating at a wavelength of 632.8 nm with vertically polarized light, was focused on the sample cell (10-mm NMR tubes, Wilmad Glass Co., of highest quality) through a temperature-controlled cylindrical quartz container (with 2 plane-parallel windows), vat (the temperature constancy being controlled to within ± 0.01 ^o^C with a heating/cooling circulator), which is filled with a refractive index matching liquid (*cis*-decalin). The isolates were filtered in an atmosphere of filtered air through a 5 μm filter (Millipore) directly into precleaned NMR tubes. The measurements were carried out at 25 ^o^C. The measurements revealed two relaxation modes, one fast and one slow mode. This suggests that there is a coexistence between single entities and aggregates in the solution.

In this work, both relaxation modes exhibit a non-exponential feature, and we have therefore fitted the correlation functions to a double stretched exponential function given by

g^1^ (t) = A_f_ exp[-(1/τ_fe_)^β^_f_] + A_s_ exp[-(t/τ_se_)^β^_s_] (1)

with A_f_ + A_s_ = 1. The parameters A_f_ and A_s_ are the amplitudes for the fast and slow relaxation mode, respectively. The variables τ_fe_ and τ_se_ are some effective relaxation times, and β (0 < β_f_ ≤ 1) and β_s_ (0 < β_s_ ≤ 1) measure of the widths of the distributions of relaxation times. The width of the distribution decreases as the stretched exponent approaches 1. The mean relaxation times are given by

$\tau_{f}=\frac{{}_{\mathrm{fe}}}{\beta_{f}}(\frac{1}{\beta_{f}})$ (2a)

$\tau_{s}=\frac{{}_{\mathrm{se}}}{\beta_{s}}(\frac{1}{\beta_{s}})$ (2b)

where Γ(β_f_^-1^) and Γ(β_s_^-1^) are the gamma functions of β_f_^-1^ and β_s_^-1^, respectively. In the analysis of the correlation function data, a nonlinear fitting algorithm (a modified Levenberg-Marquardt method) was employed to obtain best-fit values of the parameters A_f_, τ_fe_, β_f_, τ_se_, and β_s_ appearing on the right-hand side of Eq. (1). The values of β_f_ and β_s_ determined in this study were always close to 1 (β > 0.8, with one β value at 0.74). Hence, both relaxation modes are characterized by fairly narrow distributions of relaxation times.

Since both relaxation modes are diffusive and we consider dilute solutions, the apparent hydrodynamic diameter Dh (this is denoted D_h,f_ and D_h,s_ for the fast and the slow mode, respectively) is related to the mutual diffusion coefficient D via the Stokes-Einstein relationship:

$D=\frac{k_{B}T}{3{}_{0}D_{h}}$ (3)

where kB is the Boltzmann constant and η_0_ is the viscosity of the solvent at temperature T. The diffusion coefficient D is obtained from the fast or slow relaxation time through τ^-1^=Dq^2^, where the absolute value of the wave-vector, is given by q=(4πn/λ) sin (θ/2), where n is the refractive index of the medium, θ is the scattering angle, and λ is the wavelength of the light source.

S-7 Sample preparation prior to LC-MS/MS analysis

All protein preparations were performed with the use of protein LoBind tubes from Eppendorf. Reduction, alkylation and digestion were commonly performed in a thermoshaker from Grand at 600-1000 RPM (Cambridge, Cambridgeshire, UK). Unless otherwise stated, the dilutions during sample preparations were performed using water (HiPerSolv Chromanorm®) from VWR. The proteins were digested either in-solution or in-gel.

*S-7.1 Protein digestion in-solution with peptide desalting*

*Reduction and alkylation*

The lysed isolates were concentrated by a Speed-Vac^TM^ from Thermo Fisher Scientific, and the pellet was dissolved in 25 µL in a 100 mM ammonium bicarbonate (ABC, ≥99.9 %, Sigma) solution containing 6 M urea (98 %, Sigma). Prior to protein reduction, 200 mM DTT (dissolved in water for GBM isolates and 0.1 M Tris-HCl at pH 8 for BC isolates) was added to a final concentration of 9.5 mM DTT (1.25 µL). The isolates were mixed and incubated for 30 minutes at 30 °C. Next, protein alkylation was performed by adding 200 mM IAM (dissolved in the same solvent as DTT) to a final concentration of 25 mM (3.75 µL), and the isolates were mixed and incubated for 60 minutes at RT in the dark. Reduction with DTT was repeated for the BC isolates by adding 200 mM DTT to a final concentration of 28 mM (5 µL) before vortex and incubation for 30 minutes at 30 °C.

*Digestion*

The proteins in the BC isolates were initially digested by adding 0.1 µg Lys-C (from *Lysobacter enzymogens*, Sigma) dissolved in water (100 µL), followed by vortex and incubation for 120 minutes at 37 °C, before the digests were diluted with 50 mM ABC to a final concentration of 19 mM (80 µL). Proteins in the GBM isolates were only added 50 mM ABC without the Lys-C step. After the dilution, 1 µg trypsin dissolved in water was added to all isolates (50 µL). After incubating the isolates for 16 hours at 37 °C, the protease activity was terminated by adding 5 µL 50 % FA (for mass spectrometry ~98 %, Sigma) (FA/water, 50/50, v/v), giving a final concentration of 0.9 % FA.

*Peptide desalting*

Desalting of the digests was performed using ZipTip® pipette tips (Silica particles with C_18_ stationary phase) from Millipore. The ZipTip was wetted by pipetting 10 µL ACN three times and equilibrated by pipetting 10 µL of the equilibration solution (water/TFA (≥99.0 % from Sigma), 99.9/0.1, v/v) three times. Next, the digests were pipetted through the ZipTip before washing. Washing was performed by pipetting 10 µL of the washing solution (water-MeOH (VWR)/TFA, 94.9/5/0.1, v/v/v) five times. The peptides were eluted by adding 5 µL elution solution (water/ACN/TFA (69.9/30/0.1, v/v/v) for BC digests and water/ACN/TFA (69.9/30/0.1 v/v/v) for GBM digests) in a new tube and pipetting the ZipTip up and down about 20 times. The filtrate was evaporated to dryness at 30 °C by the SpeedVac. The digests were frozen at -20 °C until further use (not more than 30 days).

*S-7.2 Protein digestion in-gel*

The isolates were digested in-gel using the protocol of Shevchenko et al. [1].

*Gel electrophoresis and processing of bands from the gels*

Prior to in-gel digestion, the isolates were prepared (~3 µg protein) and run using the same procedures as with gel electrophoresis (**Section S-4.2**). BSA (3 µg, also used in BCA assays) was prepared in parallel. The gel was covered by a fixation buffer (water/MeOH-acetic acid, 40/50/10, v/v/v) overnight (18 hours at 4°C), stained with Coomassie brilliant blue (Bio-Rad) for 4 hours at RT and destained overnight with type 1 water. The gel was cut horizontally to yield four fractions from each lane; approximately 0-25 kDa, 25-70 kDa, 70-130 kDa (70-250 for BC isolates) and 130-up kDa (250-up kDa for BC isolates). The lane containing BSA was cut in the mass range of 55-70 kDa. Each fraction was sliced into cubes of ~1 mm^2^ and transferred to Protein LoBind tubes. 500 µL neat ACN was added and removed after 10 minutes of incubation and a “spin-down” of the gel pieces.

*Reduction and alkylation*

10 mM DTT in 100 mM ABC was added to cover the gel pieces and the pieces were incubated and mixed for 30 minutes at 56 °C. Next, the pieces were cooled down to RT, 500 µL neat ACN was once more added and removed after 10 minutes of incubation and “spin-down” of the gel pieces. 55 mM IAM in 100 mM ABC was added to cover the gel pieces, and the pieces were mixed and incubated in the dark for 20 minutes at RT. The step with neat ACN was repeated, however now with occasional vortexing until the coomassie dye was more or less removed from the gel pieces (about 1 hour, until the gel pieces went from being dark purple to almost white).

*Digestion and extraction*

The buffered solution of 13 ng/µL trypsin in ACN/ABC (10 mM) (10/90, v/v) was added to cover the gel pieces and allowed to rehydrate for 30 minutes on ice. Additionally 50 µL of 13 ng/µL trypsin was added to completely cover the gel pieces. The gel pieces were again left for saturation (90 minutes on ice) before adding 20 µL 10 mM ABC to ensure that the gel pieces were hold wet during the enzymatic cleavage. The tubes were placed into a GC-17A oven from Shimadzu (Kyoto, Japan) with circulating thermostat holding 37 °C. The digest was incubated overnight (≥16 hours). The peptides were extracted from the gel by adding 100 µL extraction buffer and incubation for 15 minutes in a thermoshaker. As a final step, the supernatant was collected, the digests were concentrated to dryness and kept at -20 °C until further use (≤7 days). The remaining gel pieces were also kept at -20 °C.

S-8 LC-MS/MS settings

Proteins digested in-gel were analysed in laboratory 1 and proteins digested in-solution were analysed by laboratory 2.

*S-8.1 LC-MS/MS settings with analyses in-house (laboratory 1)*

Prior to LC-MS/MS analyses, the digested isolates were thawed, dissolved in 15 µL 0.1 % water/FA (99.9/0.1, v/v) and transferred to 0.3 mL capped microvials from VWR. An EASY-nLC 1000 pump (with auto sampler) from Thermo Fisher Scientific was applied throughout the experiments.

*Columns and connections*

Precolumns (50 µm ID x 20-50 mm) and analytical columns (50 µm ID x 150 mm) were made from fused silica capillaries from Polymicro Technologies (Phoenix, AZ, USA). Packing was performed with C_18_-Accucore particles (2.6 µm beads, 80 Å pore size) from Thermo Fisher Scientific using the developed method as described in our previous study [2]. Pre- and analytical columns were additionally coupled by capillaries (i.e. fused silica capillaries of 20 µm ID from Polymicro Thechnologies), stainless steel unions (ZU1C), steel nuts (1.4 mm) and ferrules (fused silica adapters, FS1.4), all three from Vici Valco (Houston, TX, USA). The LC-column was connected to a 40 mm stainless steel nano-bore emitter (ES542, 20 µm ID) from Thermo Fisher Scientific using “Upchurch PEEK Microtight® Connector Butt” with “MicroFingertight I Fittings” and a 360 μm ID “Upchurch Microtight® Tubing Sleeve” from Sigma.

*Mobile phases and gradients*

The mobile phases consisted of water/FA (99.9/0.1, v/v) and ACN/FA (99.9/0.1, v/v) abbreviated as MP A and MP B, respectively. 10 µL of the digested isolates were picked up by the auto sampler with a flow rate of 20 µL/min. Trapping of analytes in the pre-column was performed with 100 % MP A with a maximum flow rate restricted not to reach above 500 bar (12 μL). Both the pre-column and the analytical column were equilibrated between the runs with 2- and 5 µL MP A with a maximum flow rate restricted not to reach above 600 bar. A 120 min linear gradient elution from 3-15 % MP B was used. All steps included in each run (e.g. washing) are shown in **Table S.3**

**Table S.3: The gradient elution set for the peptides in the exosome isolates, with time (minutes), duration of the gradient (minutes), flow rate (nL/min) and percentage of MP B (%).**

| **Time,**  **minutes** | **Duration of gradient,**  **minutes** | **Flow**  **nL/min** | **% B** |
| --- | --- | --- | --- |
| 0 | 0 | 130 | 3 |
| 3 | 3 | 130 | 3 |
| 123 | 120 | 130 | 15 |
| 128 | 5 | 130 | 50 |
| 130 | 2 | 173 | 80 |
| 145 | 15 | 173 | 80 |

*MS detection*

The Q-Exactive^TM^ (quadrupole orbitrap) MS was equipped with a nanoFlex nanospray ion source, both from Thermo Fisher Scientific. The capillary voltage was set to 1.8 kV and the temperature to 250 °C and 275 °C for the BC and GBM isolates, respectively. The MS was operated in data-dependent (dd) positive mode to automatically switch between MS and MS/MS acquisition. Survey full scan MS spectra (with a mass filter of *m/z* 350 to 1850) were acquired with a resolution of 70 000, automatic gain control (AGC) of 1 x10^6^ and maximum injection time of 120 ms.

For dd/MS/MS, the resolving power was set to 17 500, the AGC to 1 x10^5^ and the maximum injection time to 60 ms. The normalized collision energy was set to 28 eV, charges of 1, 7 or ≥ 8 were excluded and dynamic exclusion was set to 70.0 seconds. The method allowed sequential isolation of up to the ten most intense ions depending on signal intensity (intensity threshold 2.0 x10^4^), with isolation window of *m/z* 1.8.

*S-8.2 LC-MS/MS settings with analyses by laboratory 2*

Prior to LC-MS/MS analyses, the digested isolates were thawed and dissolved in 10 µL water/FA (99.9/0.1, v/v). An Ultimate 3000 nano ultra-HPLC system from Dionex (Sunnyvale, CA, USA) was used in combination with a MS system. The analytical column applied was an Acclaim PepMap 100 column (C18, 3 µm beads, 100 Å, 75 μm ID) of 500 mm bed length, also from Dionex.

MP A was water/FA (99.9/0.1, v/v) and MP B ACN-water-FA (90/9.9/0.1, v/v/v). The gradient elution of the analyses with time, duration of the gradient, flow rate and percentage of MP B are shown in **Table S.4.** The injection volume was 5 µL.

**Table S.4:** **The gradient elution of the analyses of exosome isolates performed by laboratory 2, with time (minutes), duration of the gradient (minutes), flow rate (nL/min) and percentage of MP B (%).**

| **Time,**  **minutes** | **Duration of gradient,**  **minutes** | **Flow**  **nL/min** | **% B** |
| --- | --- | --- | --- |
| 0 | 0 | 300 | 4 |
| 207 | 207 | 300 | 35 |
| 227 | 20 | 300 | 50 |
| 229 | 2 | 300 | 80 |

*MS detection*

The Q Exactive MS was equipped with nano-ESI source and the MS was operated in data-dependent (dd) positive mode. Survey full scan MS spectra (with a mass filter of *m/z* 400 to 1700) were acquired with a resolution of 70 000, automatic gain control (AGC) of 3 x10^6^ and maximum injection time of 100 ms.

When in dd/MS/MS the resolving power was set to 35 000 and the maximum injection time to 120 ms. The dynamic exclusion was set to 60.0 seconds. The method allowed sequential isolation of up to the ten most intense ions depending on signal intensity (intensity threshold 1.7 x10^4^) isolation window was *m/z* 2 without offset.

S-9 Data procession and protein identification with gene ontology annotation

XCalibur^TM^ (version 2.1) and Proteome Discoverer^TM^ (version 1.4.0.228) (both from Thermo Fisher Scientific) were softwares used to identify peptides and proteins. Mass spectra and chromatograms were acquired using the Xcalibur software, while protein identifications were implemented using Proteome Discoverer software. The proteins were identified using both SEQUEST and MASCOT algorithms searching the Swiss-Prot database (human taxonomy, April 2017 (20 198 entries) and June 2017 (20 205 entries) for BC- and GBM isolates, respectively)

All searches were performed setting the digestion enzyme to be trypsin with maximum one missed cleavage, fragment ion mass tolerance of 0.10 Da, and a precursor mass tolerance of 10.0 ppm. Signal to noise (S/N) threshold was set to 1.5 and the minimum ion count to 1. An automatic decoy search was performed with a false discovery rate (FDR) threshold of 0.01. Carbamidomethyl of cysteine was specified as static modification. Oxidation of methionine, acetylation of the N-terminus and deamidation of glutamine and asparagine (abbreviated Q and N) were specified as dynamic modifications. One signature peptide was selected as a requirement for protein identifications during database search. The proteins were identified as protein groups with high peptide confidence filter. All data were exported to Excel sheets and proteins identified as keratin or trypsin was removed from the list.

**References**

1. Shevchenko A, Tomas H, Havli J, Olsen JV, Mann M (2007) In-gel digestion for mass spectrometric characterization of proteins and proteomes. Nature Protocols 1 (6):2856. doi:10.1038/nprot.2006.468

2. Berg HS, Seterdal KE, Smetop T, Rozenvalds R, Brandtzaeg OK, Vehus T, Lundanes E, Wilson SR (2017) Self-packed core shell nano liquid chromatography columns and silica-based monolithic trap columns for targeted proteomics. Journal of Chromatography A 1498:111-119
