# Supplementary material for: Ultracentrifugation versus kit exosome isolation: nanoLC–MS and other tools reveal similar performance biomarkers, but also contaminations
Source: Future Sci OA. 2018 Nov 9;5(1):FSO359. doi: 10.4155/fsoa-2018-0088 (PMC6331754; doi:10.4155/fsoa-2018-0088)
Supplement: Supplementary file 4 [file fsoa-05-359-s4.pdf]

Supplemental Western Blots (uncropped raw files)

(AUTHOR INFORMATION TO BE PLACED HERE AFTER REVIEW)

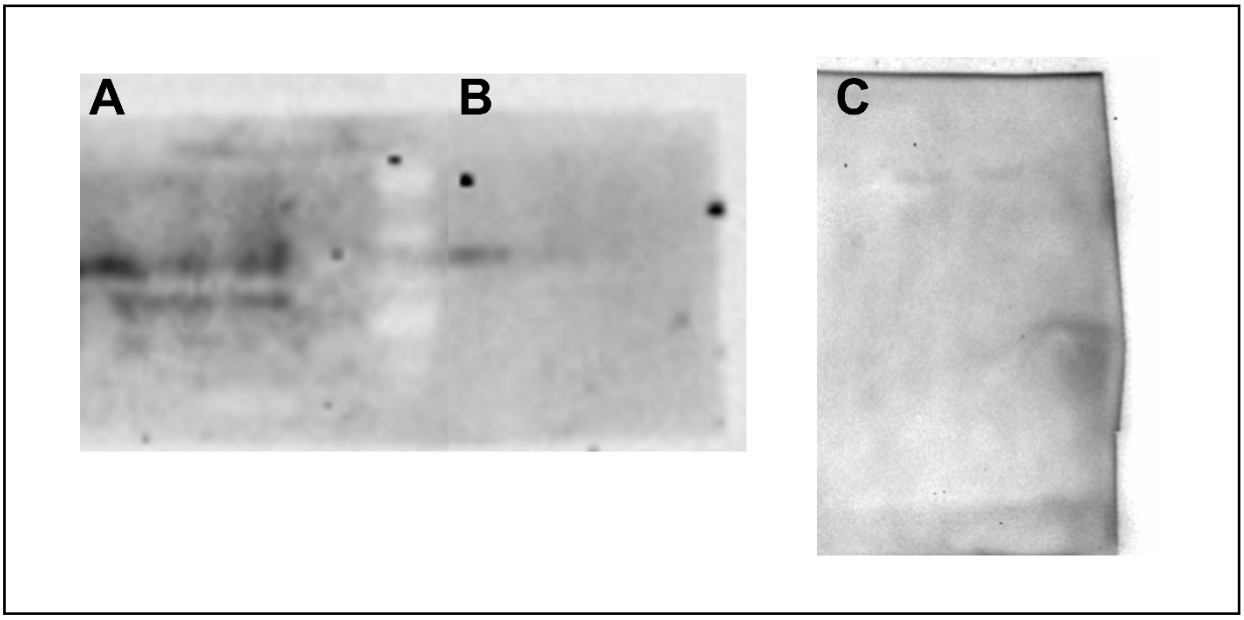

Figure WB.1 Raw files from the WB with anti-CD81 of MDA-MB-231 cell lysates (**A**, n=3) and exosome samples isolated by UC (**B**, n=2) and kit (**C**, n=3)

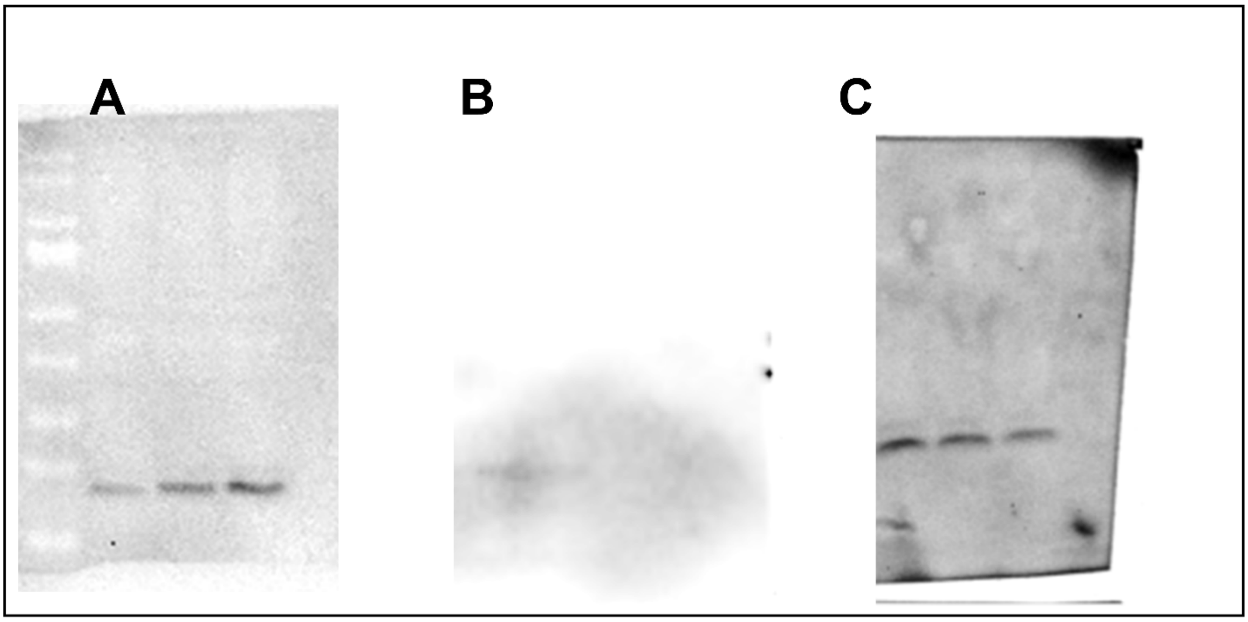

Figure WB.2 Raw files from the WB with anti-CD9 of MDA-MB-231 cell lysates (**A**, n=3) and exosome samples isolated by UC (**B**, n=2) and kit (**C**, n=3).

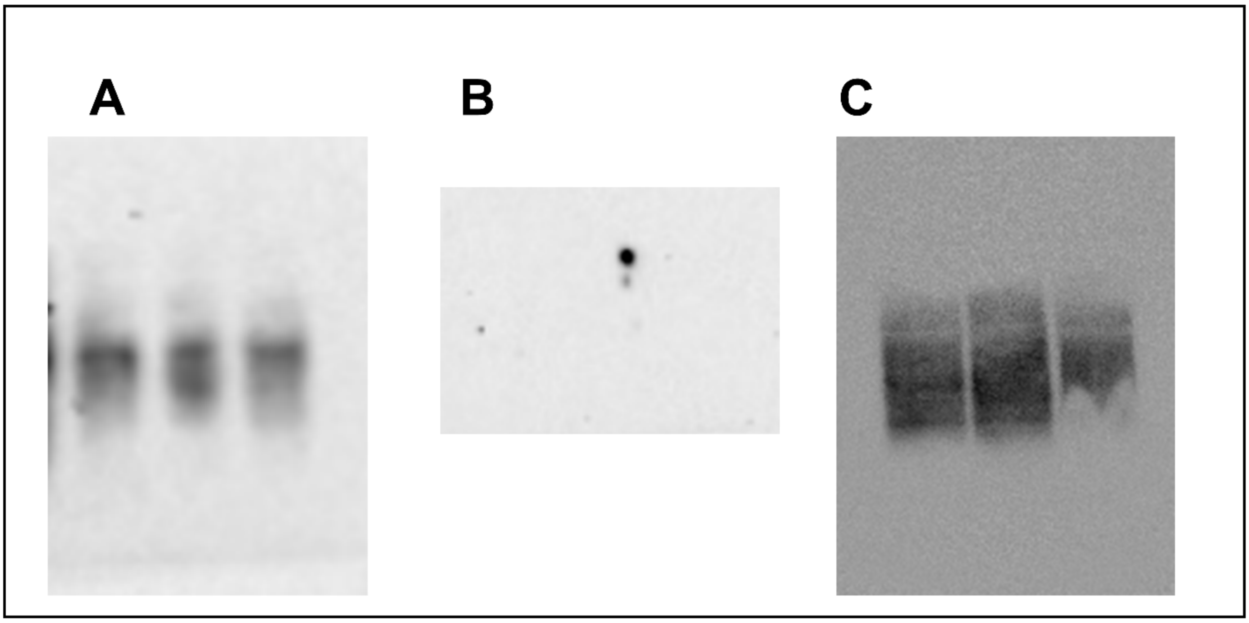

Figure WB.3 Raw files from the WB with anti-CD63 of MDA-MB-231 cell lysates (**A**, n=3) and exosome samples isolated by UC (**B**, n=2) and kit (**C**, n=3)

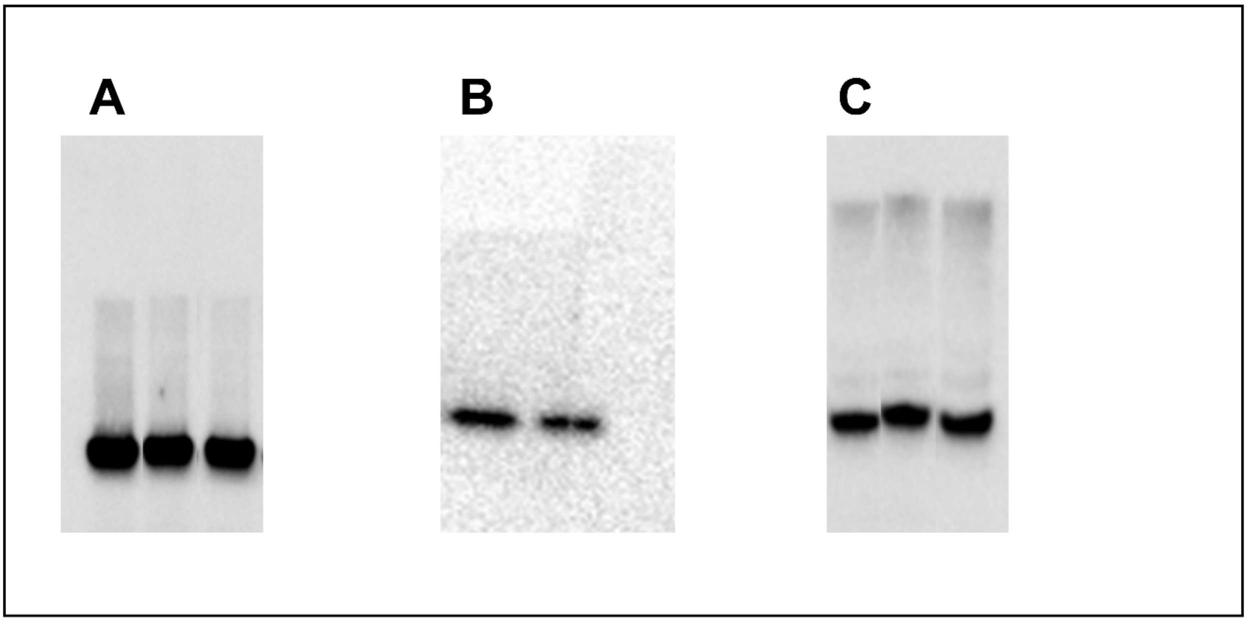

Figure WB.4 Raw files from the WB with anti-TSG101 of MDA-MB-231 cell lysates (**A**, n=3) and exosome samples isolated by UC (**B**, n=2) and kit (**C**, n=3)

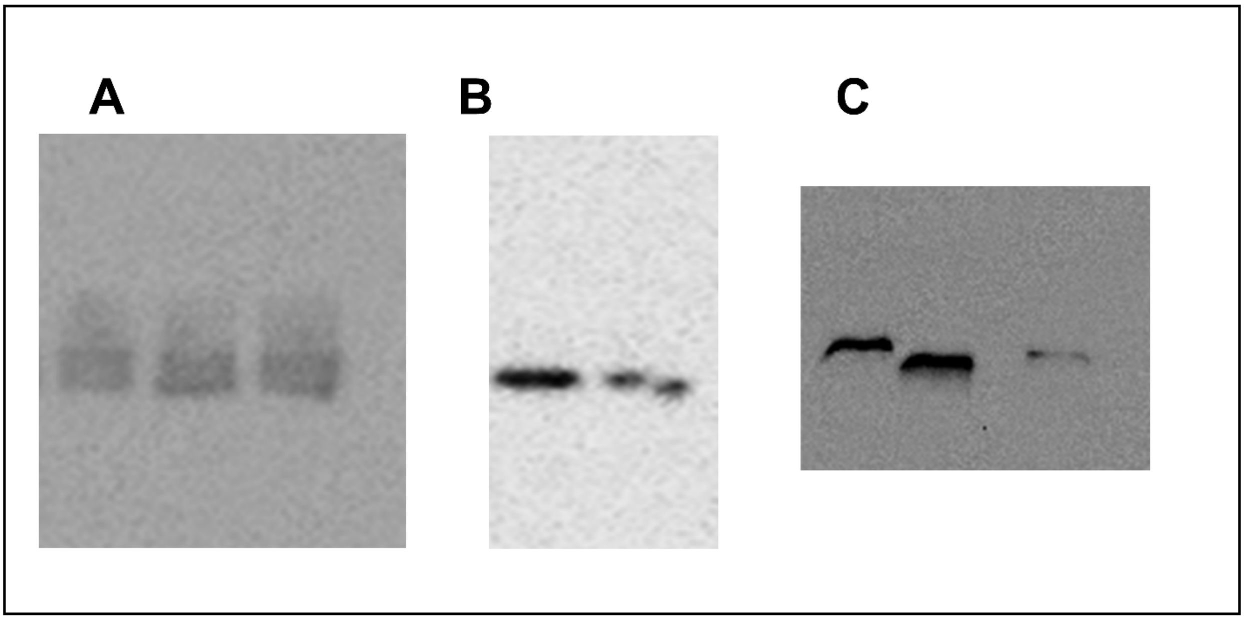

Figure WB.5 Raw files from the WB with anti-flotillin-1 of MDA-MB-231 cell lysates (**A**, n=3) and exosome samples isolated by UC (**B**, n=2) and kit (**C**, n=3)

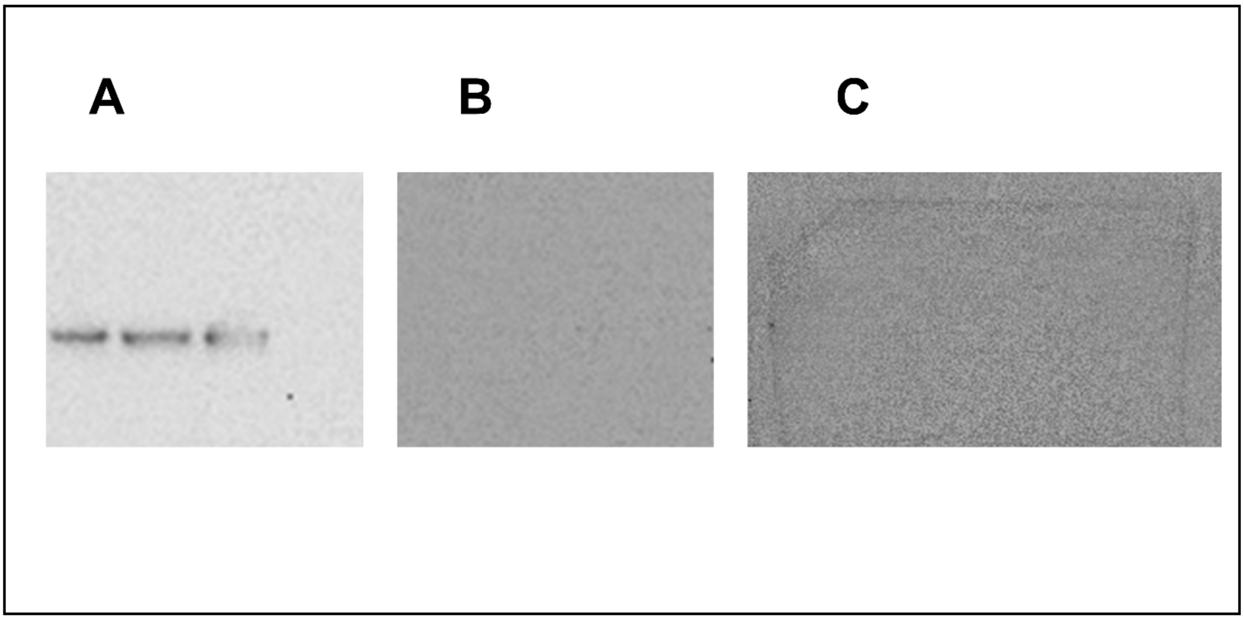

Figure WB.6 Raw files from the WB with anti-calnexin of MDA-MB-231 cell lysates (**A**, n=3) and exosome samples isolated by UC (**B**, n=2) and kit (**C**, n=3)

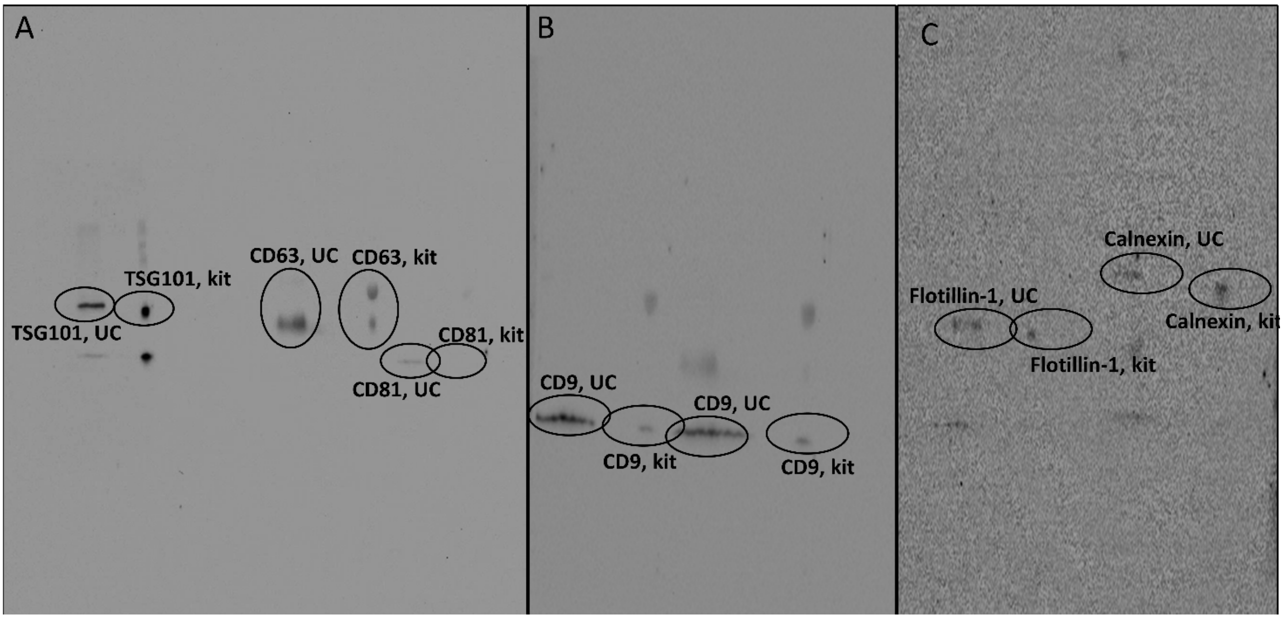

Figure WB.7 Raw files from the WB of exosome markers form GBM exosomes. **A**: WB with anti-TSG101, anti-CD63 and anti-CD81 in kit- and UC samples (n=1). **B**: WB with anti-CD9 (n=2) in kit- and UC samples. **C**: WB with anti-flotillin-1 (n=1) and anti-calnexin (n=1) in kit- and UC samples.

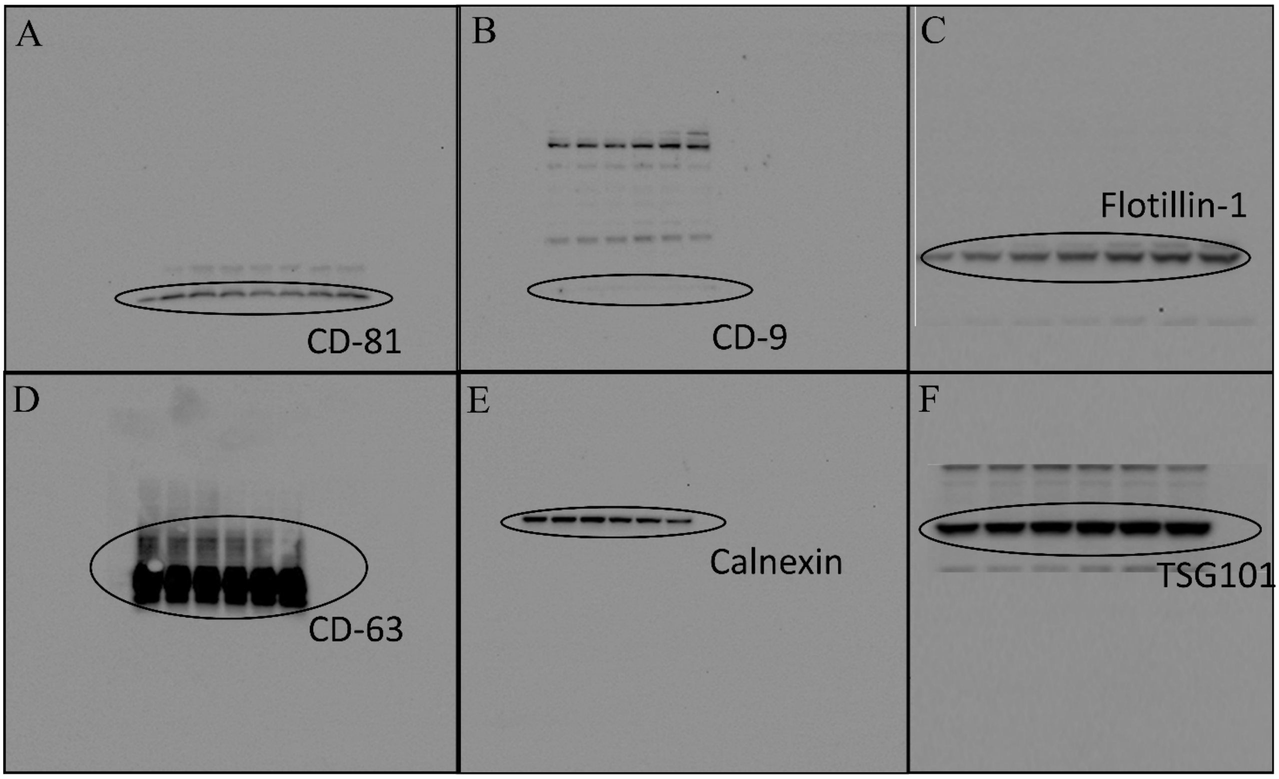

Figure WB.8 Raw files of the WB of protein markers form GBM cells (n=6). A: WB with anti-CD81. **B**: WB with anti-CD9. **C**: WB with anti-flotillin-1. **D**: WB with anti-CD63. **E**: WB with anti-calnexin. **F**: WB with anti-TSG101.

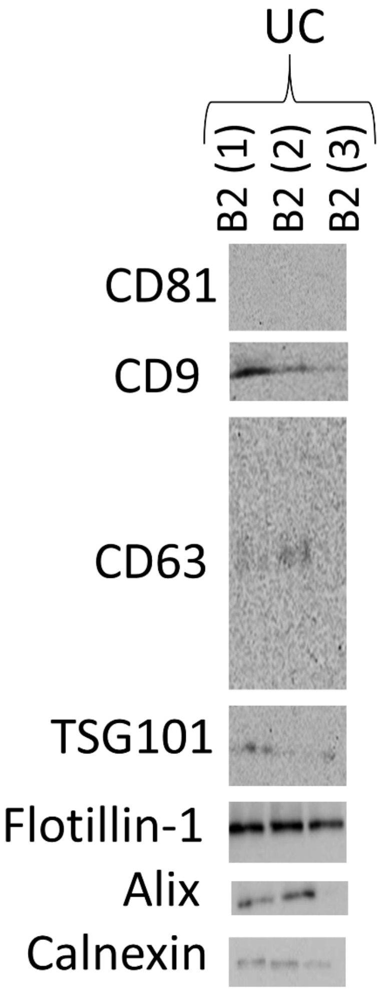

Figure WB.9 Presence of exosome markers form GBM exosomes isolated by UC (second batch). The protein markers CD81, CD9, CD63, TSG101, flotillin-1 (positive markers, +) and calnexin (negative marker, -) were targeted (n = 3). Monoclonal mouse antibodies were used for CD81, CD9, CD63, flotillin-1 and calnexin, while a polyclonal rabbit antibody was used for TSG101.
